# HIF-1A Expression in Placenta of Pregnancies Complicated with Preeclampsia and Fetal Growth Restriction

**DOI:** 10.3390/diagnostics15151843

**Published:** 2025-07-22

**Authors:** Choo Xiang Tan, Hannah Xin Yi Yeoh, Nur Aqilah Amani Mohamad Tazilan, Jonathan Wei De Tan, Nurwardah Alfian, Haliza Zakaria, Shamsul Azhar Shah, Rahana Abd Rahman, Yin Ping Wong, Geok Chin Tan

**Affiliations:** 1 Department of Pathology, Faculty of Medicine, Universiti Kebangsaan Malaysia, Kuala Lumpur 56000, Malaysia; p109401@siswa.ukm.edu.my (C.X.T.); hannahyeohxy@gmail.com (H.X.Y.Y.); amani7175@gmail.com (N.A.A.M.T.); nurwardah@hctm.ukm.edu.my (N.A.); haliza9623@ukm.edu.my (H.Z.); 2 Department of Obstetrics and Gynaecology, Faculty of Medicine, Universiti Kebangsaan Malaysia, Kuala Lumpur 56000, Malaysia; tanweide@gmail.com (J.W.D.T.); drrahana@hctm.ukm.edu.my (R.A.R.); 3 Department of Community Health, Faculty of Medicine, Universiti Kebangsaan Malaysia, Kuala Lumpur 56000, Malaysia; drsham@hctm.ukm.edu.my

**Keywords:** fetal growth restriction, HIF-1A, hypertension, placenta, pregnancy

## Abstract

**Background**: The worldwide prevalence of FGR is about 13% and can lead to various adverse perinatal outcomes, including preterm birth, stillbirth, and neonatal mortality. Hypoxia-Inducible Factor-1 (HIF-1) is an important regulator of oxygen homeostasis in humans and is crucial for placental development. The aim of this study is to determine the pattern of HIF-1A expression in placenta, and to correlate its association with preeclampsia, fetal growth restriction and adverse perinatal outcomes. **Methods**: This study comprised a total of 158 cases with 42 cases of mother having babies with fetal growth restriction (FGR), 39 cases of mother with preeclampsia (PE), 35 cases of mother with preeclampsia and fetal growth restriction and 42 controls. The expression of HIF-1A was evaluated in various placental cell types, including cytotrophoblasts, syncytiotrophoblasts, fetal endothelial cells, maternal endothelial cells, and decidual cells. **Results**: The expression of HIF-1A in placental decidual cells of mother with FGR (21/42, 50%, *p* < 0.0001), PE (25/39, 64.1%, *p* < 0.0001) and PE with FGR (12/35, 34.3%, *p* < 0.0001) were significantly increased compared to controls (1/42). Intriguingly, HIF-1A expression was significantly reduced in the placental cytotrophoblasts and syncytiotrophoblasts of mother with PE and FGR (2/35, 5.7%) compared to PE alone (11/39, 28.2%) (*p* = 0.0142). **Conclusions**: We found that increased HIF-1A expression in the nuclei of decidual cells was observed in the mothers of babies with FGR, both with and without PE. While HIF-1A expression in the cytotrophoblasts and syncytiotrophoblasts was significantly reduced between mothers with PE and mothers with PE and FGR. This suggests HIF-1A expression might play a role in the pathogenesis of FGR.

## 1. Introduction

The placenta is composed of both the fetal and maternal tissues. It supports the developing fetus. It is an essential organ that facilitates the exchange of nutrients, gases, and wastes between mother and fetus [1,2,3]. Preeclampsia (PE) resulted from impaired early placental development [3,4]. Collective evidence supports the association of hypoxia in the development of preeclampsia [5]. Both PE and fetal growth restriction (FGR) have been linked to elevated levels of hypoxia-inducible factor-1A (HIF-1A) in the human placenta, which is a major component in the cellular oxygen-sensing machinery and is also an important regulator of placental development and trophoblast differentiation [1].

Hypertension in pregnancy is an umbrella term, which encompasses chronic hypertension, gestational hypertension, PE, eclampsia, and HELLP (hemolysis, elevated liver enzymes and low platelets) syndrome [6]. Preeclampsia is a disorder of widespread vascular endothelial malfunction and vasospasm that occurs after 20 weeks of gestation [7]. The presenting features of preeclampsia includes headache, visual disturbance, abdominal pain, nausea, vomiting, proteinuria, or rapidly progressive oedema. Unfortunately, most women are asymptomatic, making the clinical evaluation of PE difficult and requiring frequent antenatal monitoring of blood pressure and urine protein level.

PE occurs in 2–8% of all pregnancies. It is one of the leading causes of maternal and perinatal mortality and morbidity worldwide [1,8,9,10]. Studies showed the key pathophysiology of preeclampsia include the placentation process, changes in the immune system, implantation, vascular remodeling, and fetal–maternal communication [9].

The Hypoxia-Inducible Factor (HIF) is a transcriptional factor that plays the role in controlling cell growth, differentiation and metabolism [1,11,12]. HIF-1 is a heterodimer made up of an oxygen regulated HIF-1A and HIF-2A subunits and a constitutively expressed HIF-1B. HIF-1 is an important regulator of oxygen homeostasis in humans and is crucial for placental development [1,13]. Under normoxic conditions, HIF-1A is rendered inactive due to its rapid break down [1,14,15,16].

The human placenta develops in a hypoxic environment during the first trimester of pregnancy due to the occlusion of uterine spiral arterioles caused by invasion of extravillous trophoblasts into the decidua, which is critical for a successful pregnancy [17,18]. This period of low oxygen tension is critical for placental and embryonic development [19] as it generates HIF that induces anaerobic processes, reduces oxygen consumption, and promotes angiogenesis, which establishes and improves blood supply [18]. Increased placental HIF-1A after 12 weeks of gestation has been found to be associated with preeclampsia in humans [20,21]. This study aims to determine the pattern of HIF-1A expression in the placenta and to correlate its association with PE, FGR, and adverse perinatal outcomes.

## 2. Materials and Methods

### 2.1. Study Design

This was a single-centre, cross-sectional, retrospective study using archival histopathological material, formalin-fixed paraffin-embedded (FFPE) tissue blocks of placenta tissue of mother with pregnancies complicated with preeclampsia and/or fetal growth restriction between 1 January 2018 and 31 December 2022. The patients’ medical records were retrieved from the integrated laboratory medical system (ILMS) in our hospital. This study included a total of 158 cases [42 cases of mother of babies with FGR, 39 cases of mother with PE, 35 cases of mother with PE and FGR, and 42 controls (mother without PE and FGR)]. The relevant clinicopathological data such as age, race, gravidity, parity, period of gestation, baby weight, and mode of delivery were obtained from the ILMS and patient medical records. This study was approved by our institutional ethics committee (approval code number: JEP-2022-585). Patient data was made anonymous with each subject assigned a coded accordingly.

### 2.2. Tissue Preparation and HIF-1A Immunohistochemistry

A full-thickness placenta tissue block was selected for immunohistochemical staining. Anti-HIF-1A antibody (EP118) (Abcam, Waltham, MA, USA) was used at a dilution 1:500. Human tonsil tissue served as a positive control tissue. Immunohistochemical staining was performed using the Evision FLEX Mini Kit, High pH (Link) (Code No. K8023, Dako Agilent, Santa Clara, CA, USA). The primary antibody was diluted to optimal dilution using Antibody Diluent, Dako REALTM (Code No. S0809, Dako Agilent Denmark, Glostrup, Denmark). Washing steps between each reagent were performed using Envision FLEX Wash Buffer 20X (Code No. K8007, Dako Agilent Denmark) diluted to a working solution with deionized water. The DAB-containing Substrate Working Solution was prepared by diluting the Envision FLEX DAB+ Chromogen with Envision FLEX Substrate Buffer (Code No. K8023, Dako Agilent Denmark).

Tissue blocks were sectioned approximately 3 µm thickness and mounted on adhesive glass slides (Platinum Pro White, Product No.: PRO-01, Matsunami Japan, Chigasaki, Japan). The slides were air-dried at room temperature overnight and then incubated on hot plate at 60 °C for 1 h. Initial deparaffinization and pre-treatment were performed in the Decloaking Chamber™ NxGen (Ref. No.: DC2012-220V, Biocare Medical California, Pacheco, CA, USA) using Envision FLEX Target Retrieval Solution, High pH (Code No. DM828, Dako Agilent Denmark) at 110 °C for 30 min, followed by cooling to room temperature for 30 min and rinsing with running tap water for 3 min. The slides were subsequently incubated with Envision FLEX Peroxidase-Blocking Reagent (Code No. DM821, Dako Agilent Denmark) for 10 min followed by a washing step.

Slides were then incubated with Envision FLEX HRP (Code No. DM822, Dako Agilent Denmark) for 30 min and followed by a washing step. Sections were then incubated with DAB-containing Substrate Working Solution for 7 min followed by a counterstain with Hematoxylin 2 (Ref. No. 7231, ThermoScientific, Waltham, MA, USA) for 5 s after the procedures have been completed, and a dehydration step with increasing alcohol solutions (80%, 90%, 100% and 100%) and 2-times Xylene. Finally, the slides were mounted using CoverSeal™-X Mounting Medium (Cat. No. FX2176, Cancer Diagnostics, Durham, NC, USA).

### 2.3. Evaluation of Immunohistochemical Staining Percentage and Intensity

The percentage and intensity HIF-1A staining were evaluated using Olympus microscope (BX40, Olympus, Japan). The location of positive staining of either in the membrane, cytoplasmic and/or nucleus was recorded. The percentages of positive cells (scored on a scale of 0–2) and staining intensity (scored on a scale of 0–3) were determined (Table 1). The percentage score and intensity score were added to obtain a total score of 0–5. Scores of 0–2 was regarded as negative, while 3–5 regarded as positive. HIF-1A expression was evaluated in the following placenta cell types: fetal endothelial cells (FEC), cytotrophoblasts (CYT), syncytiotrophoblasts (SYNT), maternal vessels endothelial cells (MVEC), and decidual cells (DC).

### 2.4. Histological Features of Maternal Vascular Malperfusion

All the cases were evaluated microscopically for histological features of maternal vascular malperfusion, which includes infarction, distal villous hypoplasia, accelerated villous maturation, increased syncytial knot formations, and decidual arteriopathy (acute atherosis, retention of smooth muscle, and mural hypertrophy), based on the criteria described by Khong and colleagues [22]. Other histological features such as acute chorioamnionitis, chronic deciduitis, chronic villitis, chronic histiocytic intervillositis, chorangioma, and single umbilical artery were also assessed using the described histological criteria of Khong et al. (2016) [22]. An Olympus microscope (BX40, Olympus, Tokyo, Japan) was used for the histological evaluation.

### 2.5. Statistical Analysis

Clinicopathological characteristics between the 2 groups were compared using a chi-square test or Fisher’s Exact test. The confident index was set at 95%. the A *p*-value of less than 0.05 is considered as statistically significant. All statistical analyses were performed using SPSS version 27 (IBM SPSS Statistics version 27, Armonk, NY, USA).

## 3. Results

### 3.1. Demographic Data

This study comprised a total of 158 cases of which 42 cases of mother of babies with FGR, 39 cases of mother with PE, 35 cases of mother with PE and FGR, and 42 controls (mother without PE and FGR). The range and average of gestational age at delivery for each group were as follows: FGR—range between 32 and 40 weeks, average of 37.5 weeks; PE—range between 32 and 39 weeks, average of 34.8 weeks; PE with FGR—range between 30 and 39 weeks, average of 34.1 weeks; and controls—range between 30 and 40 weeks, average of 37.4 weeks. The gestational age at delivery was significantly lower in the groups of mothers with PE (*p* < 0.0001) and mothers with PE and FGR (*p* < 0.0001) compared to the control group. Interestingly, mothers of babies with FGR were significantly younger than the control group (*p* = 0.040). As expected, mothers with PE and FGR were significantly older than the control group (*p* = 0.012) (Table 2).

### 3.2. HIF-1A Expression in Different Types of Placental Cells

**Figure 1:**
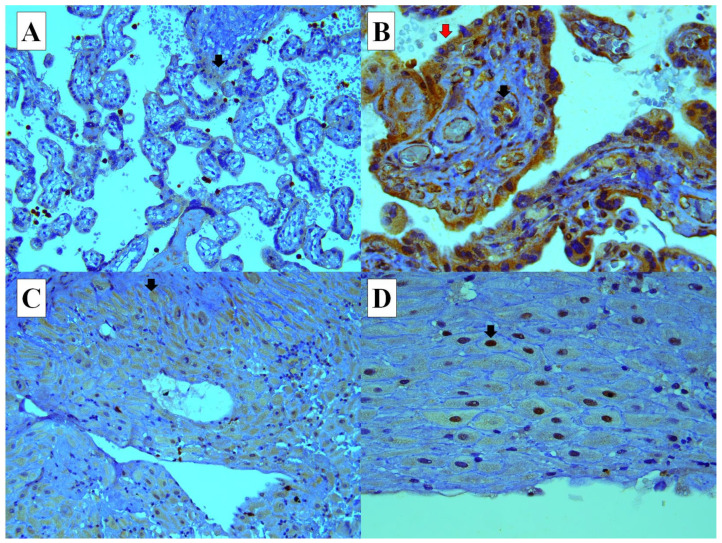
(**A**) HIF-1A expression (cytoplasmic, weak intensity) in the cytotrophoblasts and syncytiotrophoblasts (arrow) (×4). (**B**) HIF-1A expression (cytoplasmic, strong intensity) in the cytotrophoblasts, syncytiotrophoblasts (red arrow), and fetal endothelial cells (black arrow) (×40). (**C**) HIF-1A expression in the cytoplasm of the decidual cells (arrow) (×10). (**D**) HIF-1A expression in the nuclei of the decidual cells (arrow) (×40).

HIF-1A was expressed in both the nucleus and cytoplasm of the decidual cells. However, it was expressed only in the cytoplasm of cytotrophoblasts, syncytiotrophoblasts, fetal endothelial cells and maternal endothelial cells in the placenta. HIF-1A expression in the nucleus of decidual cells was significantly higher in the placentas of mothers with FGR (21/42, *p* < 0.0001), PE (25/39, *p* < 0.0001), and PE with FGR (12/35, *p* < 0.0001) compared to the control group (1/42). Notably, HIF-1A expressions in cytotrophoblasts (2/35, 5.7%, *p* = 0.0142) and syncytiotrophoblasts (2/35, 5.7%, *p* = 0.0142) were significantly reduced in the placentas of mothers with PE and FGR compared to PE alone (11/28, 28.2%) (Table 3) (Figure 1).

### 3.3. Maternal Vascular Malperfusion

We found that the histological features of maternal vascular malperfusion was significantly more frequently observed in the placenta of mothers with PE alone (22/39, 56.5%) (*p* = 0.002) and PE with FGR (28/35, 80%) (*p* < 0.0001) compared to controls (9/42, 21.4%). As expected, maternal vascular malperfusion was also more likely to be observed in the placenta of mothers with PE and FGR, compared to PE alone (*p* = 0.046). Other histological features like acute inflammation, chronic deciduitis, chronic villitis, chronic histiocytic intervillositis, chorangioma and single umbilical artery were not significantly difference between all the groups (Table 4).

## 4. Discussion

The placenta is a unique organ in pregnancy that facilitates the transport of nutrients, gases, and wastes between the mother and fetus. Impaired oxygen homeostasis could result in abnormal trophoblast differentiation and development [1]. Both PE and FGR are believed to share a common pathophysiology basis composed of endothelial dysfunction and abnormal placental implantation, resulting in insufficient trophoblast invasion and abnormal spiral artery remodelling, which eventually leads to placental ischaemia and hypoxia. A study found that women residing at high altitudes, where hypoxia is more prevalent, have a higher incidence of PE than women living at sea level [23].

One of the key mediators of the hypoxic condition is the HIF-1A. In hypoxic conditions, HIF-1A becomes stabilised and translocates into the nucleus, where it functions as a transcription factor. While HIF is induced by hypoxia, non-hypoxic stimuli such as proinflammatory factors may also cause de novo synthesis of HIF-1A [24]. Studies indicated that HIF-1A was upregulated during physical defences, innate immune responses, inflammatory responses, cytokines expression (IL-1, IL-2, IL-6, IL-8, IL-12, IL-18, and TNF), and microbial infections [25]. Overexpression of HIF-1A has been observed in various inflammatory disorders, cancer, and PE [26]. In preeclampsia, there is reduced oxygenation in the placenta due to abnormal trophoblast invasion and poor spiral artery remodelling, leading to increasing HIF-1A in the placenta. The prolonged HIF-1A expression in trophoblasts, resulted in placental disorganization, inhibition of trophoblast differentiation with endothelial dysfunction, and failed remodelling of the maternal spiral arteries [1]. We found that HIF-1A expression in the nuclei of decidual cells was significantly higher in the placenta of mother with FGR, PE, and PE with FGR compared to controls. Similarly, it has been reported by other studies [26,27,28,29].

Tal et al. (2010) demonstrated that overexpression of HIF-1A in pregnant mice produced similar key features seen in preeclampsia and fetal growth restriction, such as hypertension, proteinuria, glomerular endotheliosis, reduced fetal growth, and placental abnormalities [27]. Tianthong et al. (2020) showed that a first-trimester serum HIF-1A assessment and uterine artery Doppler could act as a screening tool to identify women at elevated risk for preeclampsia [30]. Zhang et al. (2020) demonstrated that HIF-1A was upregulated in placenta from severe preeclampsia cases and in trophoblastic HTR8/SVneo cells in the hypoxic condition. They found that hypoxia triggers nuclear translocation of HIF-1A, similar to our study observation [31].

FGR is one of the most common pregnancy complications leading to various adverse perinatal outcomes, including preterm birth, neonatal mortality, and stillbirth. It is believed that dysfunction in placental development and function, such as in angiogenesis and inadequate nutrient supply, may be the contributing factors [32]. The prevalence of FGR is about 11% in high-income countries, 10% in middle-income countries, and 19% in low-income countries. The identification of a reliable biomarker for FGR is challenging, and it has yet to be found [33]. A study showed that a low placenta growth factor (PlGF) could predict the mothers of babies with FGR [34].

In an experimental study, mice injected with an adenovirus expressing HIF-1A exhibited HIF-1A overexpression in the fetus, which resulted in intrauterine growth restriction, and their placenta was significantly smaller compared to control. The histological examination of the placenta of the mice showed extensive calcifications, vascular damage, fibrin thrombi in blood vessels, and infarction. They concluded that HIF-1A overexpression induced intrauterine growth restriction [27]. We found that HIF-1A was overexpressed in PE. HIF-1A expression was consistently increased in the nuclei of decidual cells across all three groups (FGR, PE, and PE and FGR), when compared to the control group. However, it was reduced at the cytotrophoblasts and syncytiotrophoblasts of the placentas in mothers with PE and FGR, when compared to PE alone. This finding suggests that downregulation of HIF-1A in a case of PE could have a role in the pathogenesis of FGR.

Study showed that mothers with PE have a four times higher risk of preterm delivery [35]. Our study observed the gestational age at delivery was significantly lower in the groups of mothers with PE and mothers with PE and FGR compared to the controls. Notably, we found that mothers of babies with FGR were significantly younger than the control group. Recently, a study of about 3000 women aged between 17 and 55 years old found that intrauterine growth restriction was more prevalent in the <25 years age group [36].

We found that maternal vascular malperfusion was observed in about 1/5 of placenta of mothers without PE and FGR. A study showed that 8.4% of healthy nulliparous women may have maternal vascular malperfusion changes in the placenta [37]. Freedman et al. (2023) found that the percentage of maternal vascular malperfusion ranged from 22%, 36%, and 70%, in mild, moderate, and severe PE, respectively [38]. We found that the histological features of maternal vascular malperfusion were significantly more likely to be observed in cases of PE and PE with FGR, of which it was more pronounced in the later. This finding was similar other previous studies [39,40,41]. The limitations of this study are that (1) this is a single center, retrospective study, (2) there was no correlation with serological data of HIF-1A, and (3) the sample size was small.

## 5. Conclusions

This study found that HIF-1A was significantly overexpressed in the nuclei of decidual cells of placentas in mothers of babies with FGR, both with and without PE, compared to controls. Intriguingly, its expression was significantly reduced in the cytotrophoblasts and syncytiotrophoblasts when compared between mothers with PE and mothers with PE and FGR. This suggests that downregulation of HIF-1A expression in PE may play a role in the pathogenesis of FGR. We assume that HIF-1A expression varies between cellular compartments and correlates with the severity of placental dysfunction.

## Figures and Tables

**Table 1:** Evaluation of immunohistochemical staining of HIF-1A by percentage and staining intensity.

Score	Percentage of Positive Cells	Staining Intensity
0	≤10%	Negative
1	>10%–<50%	Weak
2	≥50%–100%	Moderate
3	-	Strong

**Table 2:** Comparison of maternal demographic parameters across different groups.

Maternal Clinical Parameters	Control (*n* = 42)	FGR (*n* = 42)	*p*-Value	PE (*n* = 39) ^a^	*p*-Value	PE with FGR (*n* = 35)	*p*-Value
**Age**							
**Range (years)**	22–44	20–41	0.040 *	25–41	0.208	26–49	0.012 *
**Mean (years)**	32.6	30.5		33.8		35.5	
**SD**	4.3	4.9		4.2		5.6	
**Gestational age**							
**Range (weeks)**	30–40	32–40	0.825	32–39	<0.0001 *	30–39	<0.0001 *
**Mean (weeks)**	37.4	37.5		34.8		34.1	
**SD**	2.5	1.5		1.7		2.3	
**Ethnicity**							
**Malay**	35	32	-	32	-	25	-
**Chinese**	5	5		4		6	
**Indian**	1	2		1		3	
**Others**	1	3		2		1	
**Number of Parity**							
**Range**	1–8	1–7	<0.0001 *	1–6	<0.0001 *	1–11	0.009 *
**Mean**	3.9	2		2.4		2.8	
**SD**	1.6	1.5		1.4		2.0	

SD—standard deviation, FGR—fetal growth restriction, PE—pre-eclampsia; * *p*-value of <0.05 is considered as significant; ^a^ Two cases were unsure of date.

**Table 3:** Comparison of HIF-1A expression in the various types of placental cells.

Types of Placental Cells	HIF-1A Expression	Control (*n* = 42) (%)	FGR (*n* = 42) (%)	*p*-Value	Pre-Eclampsia (*n* = 39) (%)	*p*-Value	Pre-Eclampsia with FGR (*n* = 35) (%)	*p*-Value (C vs. PEF)	*p*-Value (PE vs. PEF)
Cytotrophoblast	Positive	6 (14.3)	17 (40.5)	0.0134 *	11 (28.2)	0.1732	2 (5.7)	0.2798	0.0142 *
	Negative	36 (85.7)	25(59.5)		28 (71.8)		33 (94.3)		
Syncytiotrophoblast	Positive	8 (19.1)	17 (40.5)	0.0551	11 (28.2)	0.4330	2 (5.7)	0.1009	0.0142 *
	Negative	34 (80.9)	25 (59.5)		28 (71.8)		33 (94.3)		
Fetal endothelial cells	Positive	0 (0)	5 (11.9)	0.0551	5 (12.8)	0.0225 *	2 (5.7)	0.2033	0.4350
	Negative	42 (100)	37 (88.1)		34 (87.2)		33 (94.3)		
Maternal endothelial cells	Positive	2 (4.8)	9 (21.4)	0.0485 *	7 (18.0)	0.0808	0 (0)	0.4976	0.0123 *
	Negative	40 (95.2)	33 (78.6)		32 (82.0)		35 (100)		
Decidual cells (cytoplasmic staining)	Positive	21 (50.0)	30 (71.4)	0.0732	11 (28.2)	0.0683	14 (40.0)	0.4912	0.3308
	Negative	21 (50.0)	12 (28.6)		28 (71.8)		21 (60.0)		
Decidual cells (nuclear staining)	Positive	1 (2.4)	21 (50.0)	<0.0001 *	25 (64.1)	<0.0001 *	12 (34.3)	0.0003 *	0.0193 *
	Negative	41 (97.6)	21 (50.0)		14 (35.9)		23 (65.7)		

HIF-1A—Hypoxic inducible factor 1 alpha, FGR—Fetal growth restriction, C—Control, PEF—Pre-eclampsia with fetal growth restriction, * *p*-value of <0.05 is considered by statistically significant.

**Table 4:** Comparison of the placental histological features between the controls, fetal growth restriction, preeclampsia and preeclampsia with fetal growth restriction.

Placental Histological Features ^a^	Control (*n* = 42) (%)	FGR (*n* = 42) (%)	*p*-Value	PE (*n* = 39) (%)	*p*-Value	PE with FGR (*n* = 35) (%)	*p*-Value
Maternal vascular malperfusion							
Positive	9 (21.4)	11 (26.2)	0.798	22 (56.4)	0.002 *	28 (80.0)	<0.0001 *
Negative	33 (78.6)	31 (73.8)		17 (43.6)		7 (20.0)	
Acute inflammation							
Positive	6 (14.3)	12 (28.6)	0.183	6 (15.4)	1.0	3 (8.6)	0.498
Negative	36 (85.7)	30 (71.4)		33 (78.6)		32 (91.4)	
Chronic deciduitis							
Positive	2 (4.8)	0 (0)	0.494	0 (0)	0.494	0 (0)	0.498
Negative	40 (95.2)	42 (100)		39 (100)		35 (100)	
Chronic villitis							
Positive	1 (2.4)	0 (0)	1.0	0 (0)	1.0	0 (0)	1.0
Negative	41 (97.6)	42 (100)		39 (100)		35 (100)	
Chronic histiocytic intervillositis							
Positive	0 (0)	1 (2.4)	1.0	0 (0)	1.0	0 (0)	1.0
Negative	42 (100)	41 (97.6)		39 (100)		35 (100)	
Chorangioma							
Positive	0 (0)	1 (2.4)	1.0	0 (0)	1.0	0 (0)	1.0
Negative	42 (100)	41 (97.6)		39 (100)		35 (100)	
Single umbilical artery							
Positive	0 (0)	0 (0)	1.0	1 (2.6)	0.482	0 (0)	1.0
Negative	42 (100)	42 (100)		38 (97.4)		35 (100)	
No significant pathology	24 (57.1)	18 (42.9)		11 (28.2)		4 (11.4)	NA

FGR—fetal growth restriction, PE—pre-eclampsia, * *p*-value of <0.05 is considered as significant, ^a^ Some cases may have 2 or more histological features.

## Data Availability

Data of this study is presented in the manuscript.

## Abbreviations

The following abbreviations are used in this manuscript:

PE	Preeclampsia
FFPE	Formalin-fixed paraffin-embedded
FGR	Fetal Growth Restriction
HIF-1A	Hypoxia-Inducible Factor-1 Alpha
HELLP	Hemolysis, Elevated Liver Enzymes, and Low Platelets
